# Addition of Zn during the phosphine-based synthesis of indium phospide quantum dots: doping and surface passivation

**DOI:** 10.3762/bjnano.6.127

**Published:** 2015-06-01

**Authors:** Natalia E Mordvinova, Alexander A Vinokurov, Oleg I Lebedev, Tatiana A Kuznetsova, Sergey G Dorofeev

**Affiliations:** 1 Department of Chemistry, Moscow State University, 1 building 3 Leninskie Gory, Moscow 119899, Russia; 2 Laboratoire CRISMAT, UMR6508, CNRS-ENSICAEN, 6 boulevard Marechal Juin, Caen 14050, France

**Keywords:** core–shell nanoparticles, doped semiconductor nanocrystals, InP(Zn) quantum dots, luminescence, zinc

## Abstract

Zinc-doped InP(Zn) colloidal quantum dots (QDs) with narrow size distribution and low defect concentration were grown for the first time via a novel phosphine synthetic route and over a wide range of Zn doping. We report the influence of Zn on the optical properties of the obtained quantum dots. We propose a mechanism for the introduction of Zn in the QDs and show that the incorporation of Zn atoms into the InP lattice leads to the formation of Zn acceptor levels and a luminescence tail in the red region of the spectra. Using photochemical etching with HF, we confirmed that the Zn dopant atoms are situated inside the InP nanoparticles. Moreover, doping with Zn is accompanied with the coverage of the QDs by a zinc shell. During the synthesis Zn myristate covers the QD nucleus and inhibits the particle growth. At the same time the zinc shell leads to an increase of the luminescence quantum yield through the reduction of phosphorous dangling bonds. A scenario for the growth of the colloidal InP(Zn) QDs was proposed and discussed.

## Introduction

Colloidal quantum dots (QDs) based on III–V materials are promising objects for fundamental research as well as for practical application. In particular, such QDs could be successfully used in biomedicine, in the production of QD-based LEDs, solar cells and sensors [1–4]. This is because of their relatively large excitonic Bohr radius and the lower toxicity in comparison to the widely used II–VI compounds [5]. For the practical application QDs should meet certain requirements: low defect concentration, narrow size distribution, and physico-chemical stability. The synthesis of these QDs should be reproducible, possibly simple and safe. Despite the advantages over II–VI materials that are typically used in these fields, the more covalent III–V materials are difficult to prepare because of the lack of suitable precursors. Commonly used in the preparation of III–V materials, organometallic precursors are not stable and can form complexes with the solvents leading to a decline in the quality of the prepared QDs [5]. Therefore, the search and the development of effective synthetic approaches, satisfying the conditions mentioned above, is a crucial point to obtain high quality III–V QDs.

Among the QDs based on III–V materials InP colloidal QDs have gained the most attention due to their stability and the most intensive luminescence in the visible and near-IR spectral regions. There are several synthetic approaches to obtain InP QDs [6–10]. One of the most commonly used methods nowadays includes the thermal decomposition of sililphosphides [11]. This is a quite complicated method because of the reactivity and inflammability of such substances. Recently, we developed the simplest way to date to produce such material by using phosphine (PH_3_) as a source of phosphorus [6] and indium carboxilates as a source of indium with various carbonic acids as surfactants in nonpolar solvents. This method leads to relatively narrow particle size distributions with mean diameters of about 1–7 nm, a high crystallinity of the nanoparticles and the temporal stability of the optical properties.

It is well established that the doping of III–V QDs creates an opportunity to produce materials with new optical properties that vary depending on the dopant type. This opportunity has promoted the development of synthetic methods for incorporating dopants into InP QDs. There are some efforts focused on the incorporation of Mn, Cu, and Eu [12–14] into InP QDs. At the same time, Zn, which has completely filled 3d and 4s orbitals exhibits a behavior identical that is more similar to that of the p-metal In than other d- and f-elements. This should result in a more stable and deep incorporation of Zn into InP QDs. The ionic radii of In^3+^ and Zn^2+^ have very close values (0.080 nm and 0.074 nm, respectively) [15]. Thus, Zn is one of the most important p-type dopants in volume InP. The presence of Zn in InP QDs is usually regarded in the context of being covered with ZnSe [16] or ZnS [17] shells, which drastically improves the optical properties of the QDs.

In this paper we discuss the contribution of Zn to the improvement of the optical properties of InP QDs synthesized through a phosphine-based synthetic route for the first time and over a wide range of Zn doping. We propose a mechanism for the introduction of Zn in the QDs and show that two parallel processes occur: the incorporation of Zn atoms into the InP lattice, which leads to the formation of Zn acceptor levels and a luminescence tail in red region of the spectra; and a zinc shell, which leads to an increase of the PL intensity.

## Results and Discussion

**Figure 1:**
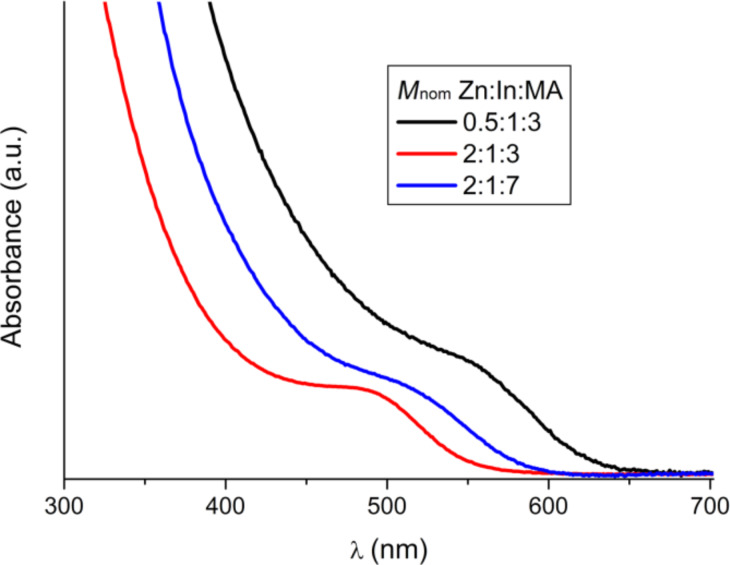
UV–vis absorption spectra of Zn-doped InP QDs.

As a result of synthesis a number of different colored solutions were obtained: yellow, orange and red. It was expected that the addition of excess myristic acid may result in a better stabilization of the QDs if any In(Zn)P alloy could be formed [18], thus enhancing the optical properties of the QDs. However, the excess myristic acid has a detrimental effect on the optical properties: The samples exhibit a more diffused absorption peak for both small and large amounts of Zn precursor (Figure 1). A high polydispersity of the samples is confirmed by TEM. The luminescence intensity for the samples with excess myristic acid is lower. Moreover, the excess myristic acid contaminates the samples and rendering them too viscous and difficult to purify.

**Figure 2:**
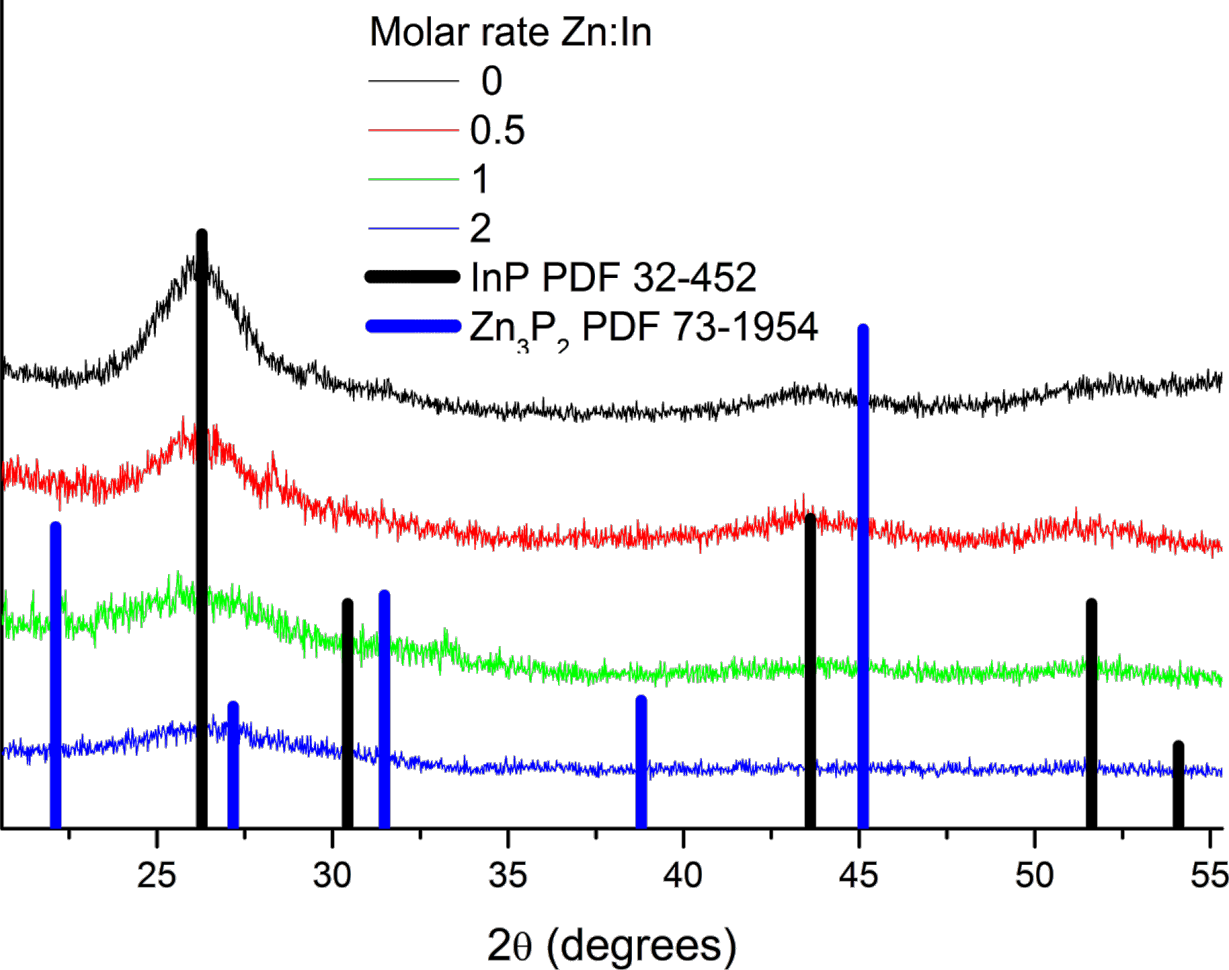
Experimental X-ray powder diffractogram for synthesized InP QDs with different amounts of Zn dopant.

**Figure 3:**
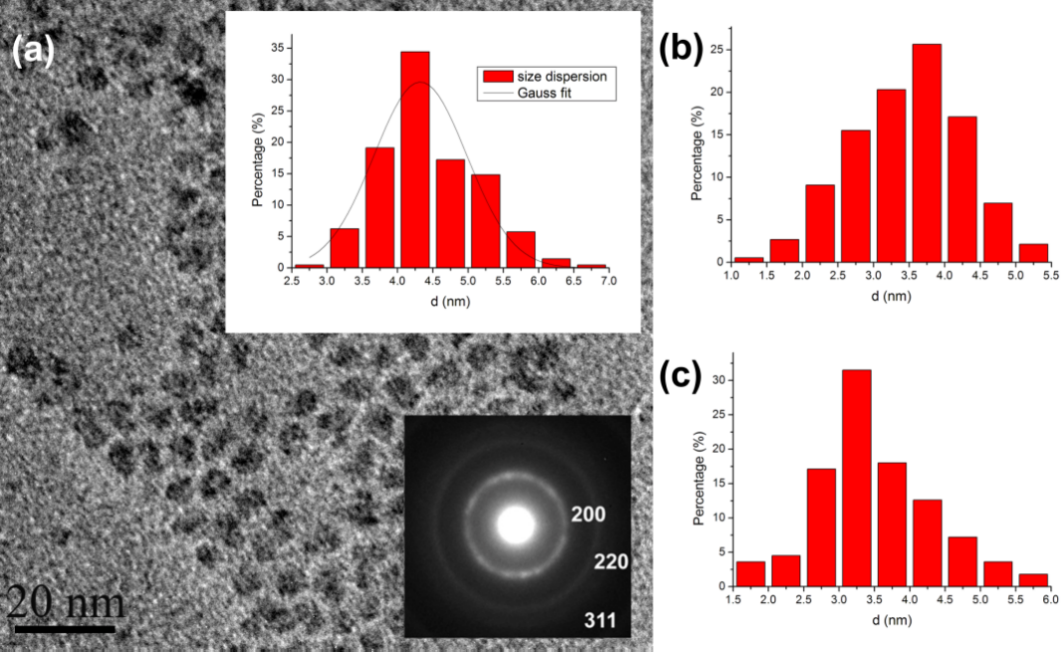
(a) Bright-field low-magnification TEM image of non-doped InP QDs and its number-weighted size distribution (upper insert). Ring electron diffraction pattern (lower insert) confirming zinc blende structure of InP; (b) Number-weighted size distribution of the sample with *M*_nom_ = 0.5; (c) Number-weighted size distribution of sample with *M*_nom_ = 1.

Figure 2 shows the powder XRD patterns of the InP nanocrystals with different Zn amounts added during the synthesis. The XRD pattern show a clear zinc blende structure of InP (*F*−43*m* space group No. 216, *a* = 5.868 Å). The reflections were indexed as (111), (220) and (311) planes. Increasing the Zn amount leads to broader peak shapes, which suggests a decrease of the QDs size. This assumption was also confirmed by TEM investigation (Figure 3). The diameter of the non-doped QDs calculated from XRD is approximately 3.8 nm. For *M*_nom_ = 0.5 the diameter is 3.3 nm, for *M*_nom_ = 1 it is 2.6 nm, and for *M*_nom_ = 2 it is 2.4 nm, where *M*_nom_ is the Zn:In molar ratio in the reaction mass.

**Figure 4:**
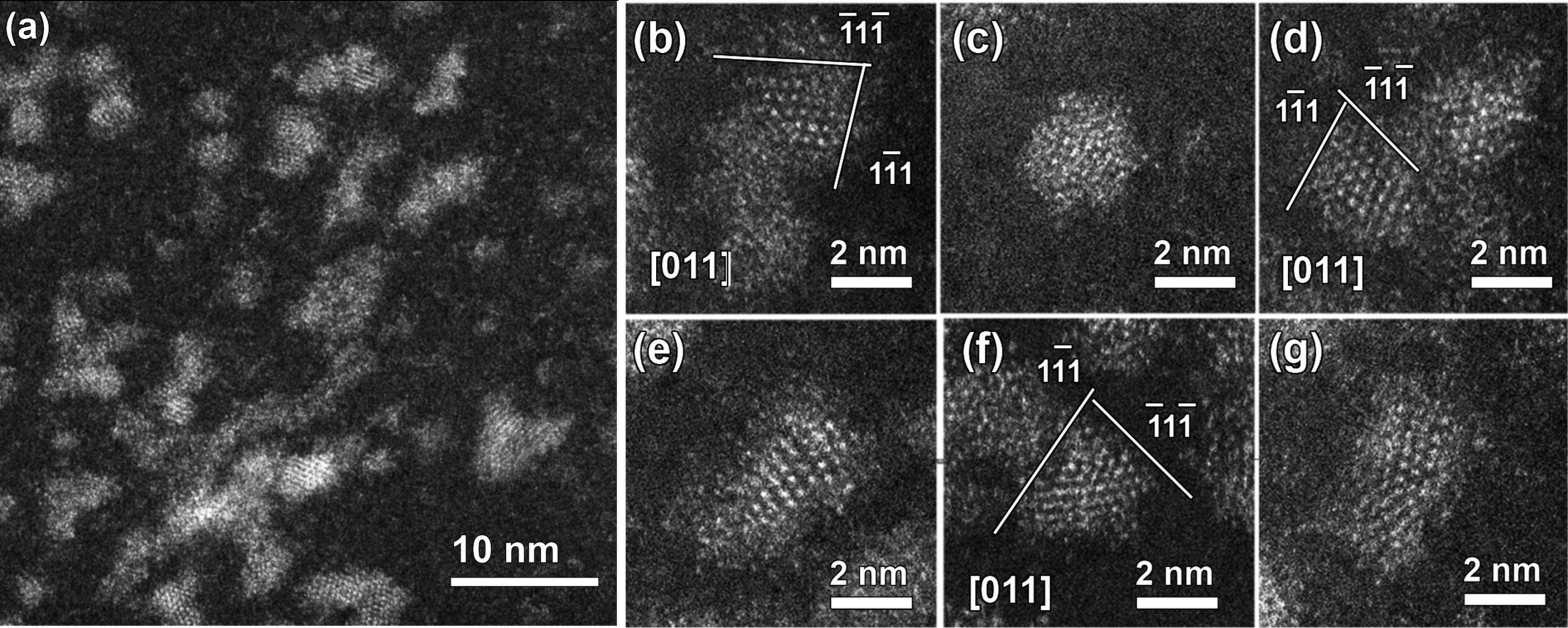
(a) Low-magnification HAADF-STEM images of Zn/InP QDs and (b–g) selected high-resolution images of single QDs along different zone axes. Notice faceting of the core and the presence of a disordered shell.

**Figure 5:**
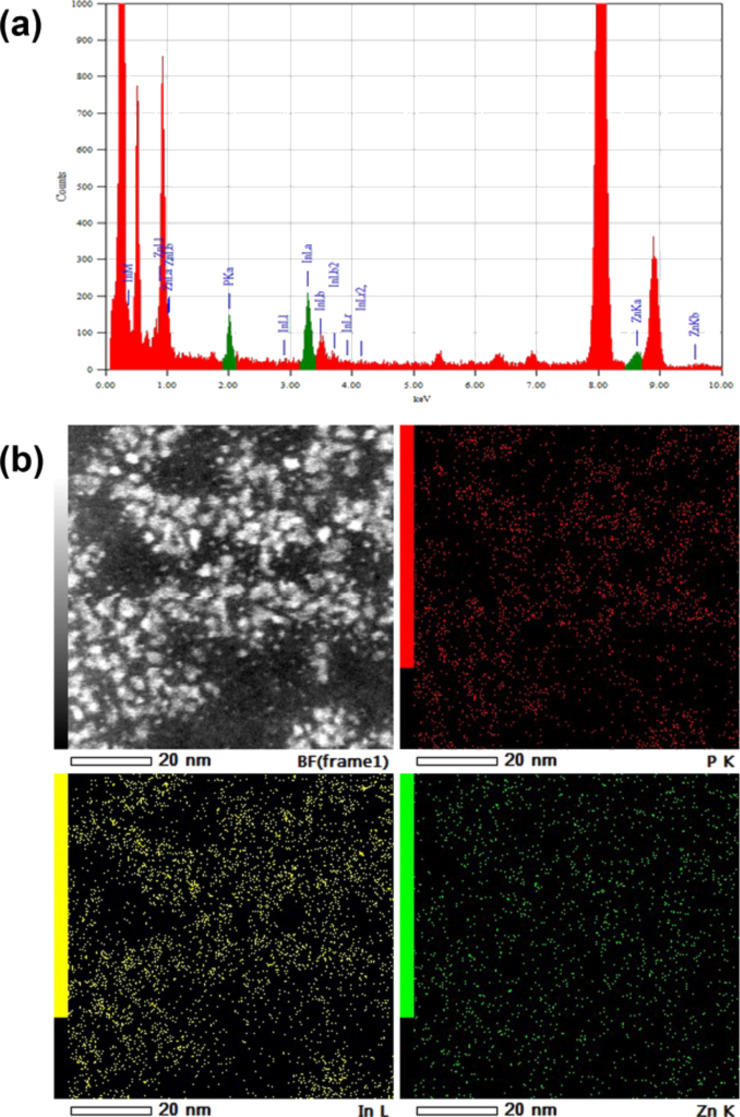
(a) EDX spectrum taken from the area of several tens of ODs; (b) HAADF-STEM image and elemental mapping of Zn/InP sample.

In order to investigate the fine microstructure of the Zn/InP QDs, and in particular the morphology, size, and defect structure of the nanoparticles as well as the Zn distribution, transmission electron microscopy was applied. The main results of the study are shown in Figure 3, Figure 4 and Figure 5.

Figure 3a shows a low-magnification TEM image of non-doped InP QDs and the corresponding electron diffraction (ED) pattern. As can be seen from the typical low-magnification TEM images in Figure 3a the prepared QDs are of almost spherical shape and the mean particle diameter is about 3–6 nm. The corresponding ED pattern exhibits distinct ring patterns, typical for the clustering of relatively small and randomly oriented QDs. It also reveals an overall high crystallinity and structural homogeneity of the nanoparticles. The rings of the ED pattern can be completely indexed based on the cubic InP structure (*F*−43*m*, space group No. 216, *a* = 5.868 Å), which is in agreement with the XRD results. No extra rings associated with secondary phase or amorphous structure has been detected.

It should be noticed that in the low-magnification TEM image in Figure 3a, the QDs can be seen as black patches, which consist of a crystal core and a surrounding organic layer. This amorphous shell consists of myristic acid and some indium myristate in the case of non-doped QDs [19] and presumably zinc myristate in case of Zn-doped QDs. The mean diameter of particles calculated from the TEM images is bigger, than that calculated from XRD because the organic layer does not contribute to the X-ray coherent scattering region. This discrepancy can be explained by fact that the contrast in bright-field low-magnification TEM is a mass-thickness contrast, which arises from Rutherford elastic scattering of electrons, rather than a diffraction or an amplitude contrast in the case of dislocations and high resolution imaging. Thus, the amorphous shell will be the basis of the main contrast in Figure 3a and, correspondingly, of the mean size of the QDs. Therefore, the change of the particle size, which depends on the Zn amount, is noticeable in the TEM images as well: for non-doped QDs *d*_mean_ ≈ 4.3 nm (Figure 3a), for QDs with *M*_nom_ = 0.5 *d*_mean_ ≈ 3.7 nm (Figure 3b) and for QDs with *M*_nom_ = 1 *d*_mean_ ≈ 3.3 nm (Figure 3c). The dependence of the particles size on the Zn amount suggests that Zn takes part in the formation of the QDs and inhibits their growth.

In order to determine the real size and structure of the core of the doped InP QDs as well as the doping distribution within the sample, high angle annular dark field scanning TEM (HAADF-STEM), the so-called *Z*-contrast was applied. The incoherent image uses high angle scattering, which leads to a strong atomic number contrast (proportional to *Z*^2^), and also makes simultaneous EDX mapping. Therefore, the contrast in HAADF-STEM image is roughly proportional to the square of the atomic number, making it possible to detect even single atoms in high resolution HAADF-STEM image (Zn = 30, In = 49, P = 15). Figure 4 shows representative HAADF-STEM images of Zn-doped InP QDs. In the low-magnification HAADF-STEM image (Figure 4a), the size of the QDs is close to that of the bright-field TEM images (Figure 3). However, upon close inspection using high resolution HAADF-STEM (Figure 4b–g), the core–shell structure of Zn/InP QDs can be clearly distinguished and confirmed.

The particles in Figure 4b–g definitely exhibit a core–shell structure with a core diameter approximately about 2 nm, with mainly {111}-type surface facets (Figure 4b,d,f). The shape of the majority of the NPs is almost spherical. However, some of the NPs exhibit an elongated shape (Figure 4e,g). The core of the NPs exhibits strong bright contrast, which correlates well with the composition of QDs with the In atoms having a larger *Z*. On the other hand, a close inspection of HAADF-STEM images, and in particular shell images, revealed two distinct features. The first feature is that the shell is less bright than the core. The second feature is that some of the dots in the shell corresponding to single atoms are darker than the atoms in the InP core. This allows us to suggest the presence of some Zn atoms in the shell. EDX analysis confirmed that the QDs consist of In and P with some Zn (Figure 5a). HAADF-STEM images and elemental mapping of InP(Zn) QDs revealed a homogeneous distribution of Zn all over the sample (Figure 5b). Taking into account the EDX mapping data (Figure 5a) and the results of the high-resolution HAADF-STEM studies, particularly the contrast of the shell, we can conclude that Zn atoms are mainly located at the surface of the InP QDs.

**Figure 6:**
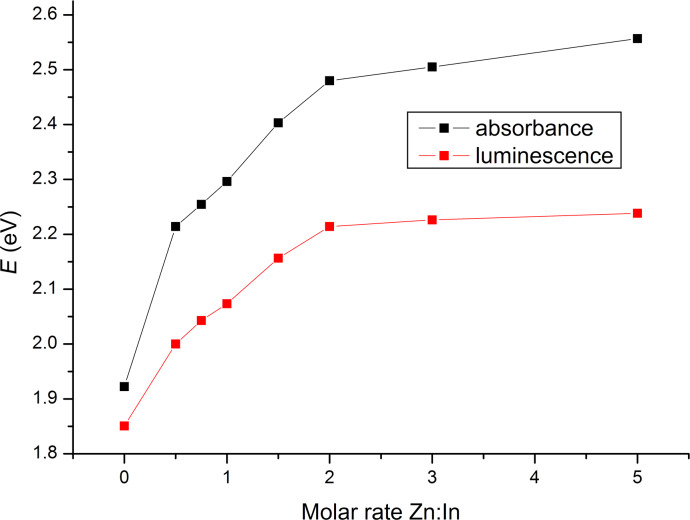
Dependence of UV–vis absorption spectra and PL peak position on *M*_nom_.

With increasing *M*_nom_ an increasing blue shift of the UV–vis absorption spectra and the PL spectra is observed. This fact also points out to a decreasing of the QDs size. The dependence of the peak position on *M*_nom_ is shown in Figure 6. One can clearly see that both, position of absorption and luminescence peak, shift towards shorter wavelengths up to *M*_nom_ = 2 and then reach a steady level. Another evidence of the correlation of Zn amount and QDs size is the Stokes shift (the red shift of the emission spectra with respect to the absorption spectra), which increases in our case with increasing Zn amount. The Stokes shift is commonly observed in semiconductor QDs and is a function of the QDs radius. As the diameter increases the Stokes shift decreases and disappears above a certain diameter [20]. The same dependence is observed for the QDs described here (Figure 6).

**Figure 7:**
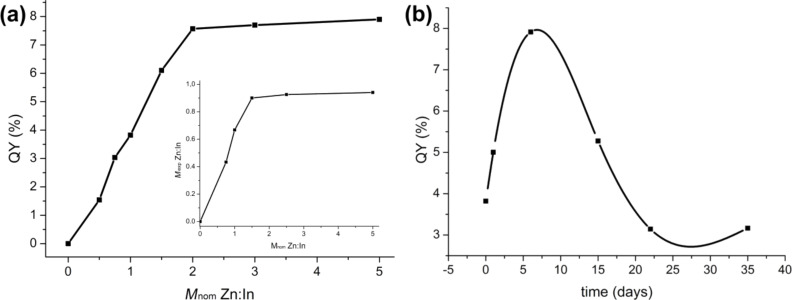
(a) Dependence of QY on *M*_nom_; (insert) dependence of *M*_exp_ on *M*_nom_, obtained from X-ray fluorescence analysis; (b) temporary evolution of luminescence intensity of samle with *M*_nom_ = 1.

Usually, non-doped QDs exhibit very low PL efficiencies immediately after preparation, i.e. the quantum yield (QY) is below 0.5%. This is because of the numerous dangling bonds present on the surface of the QDs. QY slightly increases up to 1–2% due to the oxidation of the nanocrystal surface under exposure to air [19]. However, the presence of zinc during the synthesis leads to an increase of QY in the particular case described here. The more zinc is accepted by the QDs the larger is QY. At the same time, starting from the double excess of zinc (*M*_nom_ = 2) the QY does not change any further and also reaches a steady level of about 7.5%. The dependence of QY on the Zn amount was obtained right after synthesis for all samples (Figure 7a). This significant improvement of the luminescence intensity is similar to that of one effective post-synthesis treatment, namely the covering with a shell of another semiconductor material [16–17,21]. The coating with a semiconductor shell can reduce the trap states for charge carriers and isolate the core from environmental oxygen and thus improves the PL efficiency and stability of InP QDs. Our experimental data show that in our case a covering of the particles with zinc myristate occurs. During the synthesis zinc myristate covers the nucleus and prevents the particle growth and at the same time leads to an increase of the luminescence intensity through the reduction of phosphorus dangling bonds.

The real Zn amount in the sample was measured by using X-ray fluorescence (XRF) spectroscopy. The experimentally determined Zn:In molar ratio (*M*_exp_) compared to the nominal Zn:In molar ratio is shown in Figure 7a (insert). Immediately after synthesis a white precipitate, which is insoluble in nonpolar solvents was centrifuged out. According to XRF spectroscopy, this precipitate consists mostly of zinc myristate. Apparently, the amount of Zn after synthesis is less than the one that was put into the synthesis. *M*_exp_ increases with increasing *M*_nom_, but above a threshold of *M*_nom_ = 2 the increase stops. This point matches well with the beginning plateaus of UV–vis absorption, luminescence peak position and QY. So we can observe that the optical properties are closely related to the amount of Zn in the sample and that there is a maximum amount of Zn that can be introduced into the QDs. It should be noted that the highest amount of Zn in the samples, *M*_exp_ ≈ 0.9, cannot be accounted for by doping alone. Thus, the surface-absorbed Zn atoms are taken into account. These surface Zn-atoms do not form a separate phase, because there is no second phase found by XRD. This Zn shell is not strongly bound to the surface of the QDs and degrades over time.

Figure 7b shows the luminescence intensity evolution after synthesis for the sample with *M*_nom_ = 1, which is typical for all samples. Initially, QY slightly increases due to the oxidation of the nanocrystal surface, comparable to the case of non-doped QDs. After that, the competing process of shell degradation leads to a significant decrease of QY. This process is accompanied by the formation of a white precipitate (Zn myristate) and a decrease of *M*_exp_ (according to XRF spectroscopy).

**Figure 8:**
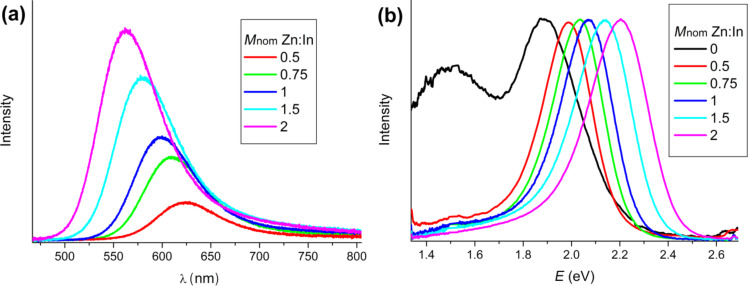
(a) PL spectra of synthesized QDs; (b) normalized PL spectra in energy coordinates.

We suppose that the formation of the Zn shell occurs during the synthesis and it is an important preliminary step for the covering with semiconductor shells (ZnSe or ZnS [16–17]). But there is another important process, namely the doping of QDs with Zn. Bulk Zn doping of InP requires very reactive precursors, high temperatures and a long reaction time [22]. Therefore, we cannot expect that a lot of Zn atoms could be incorporated into the InP crystal lattice. Nevertheless, the optical properties of the synthesized QD testify that Zn-doping certainly took place. Obviously, the PL peaks exhibit asymmetrical shape (Figure 8a) with a so-called tail of luminescence in the red region of the spectra. Figure 8b shows the normalized PL spectra of samples with different amounts of Zn. The spectrum of non-doped QDs exhibit a noticeable peak related to surface defects. Zn atoms have a great influence on the surface defects, i.e., already a small amount of Zn on the surface of the QDs results in fewer surface dangling bonds, which reduces the defect peak and enhances the excitonic peak. However, the tail does not completely disappear and we suggest that it is not related to surface defects but to Zn-doping. Furthermore, we can clearly see that the form of the spectra hardly changes with increasing *M*_nom_.

**Figure 9:**
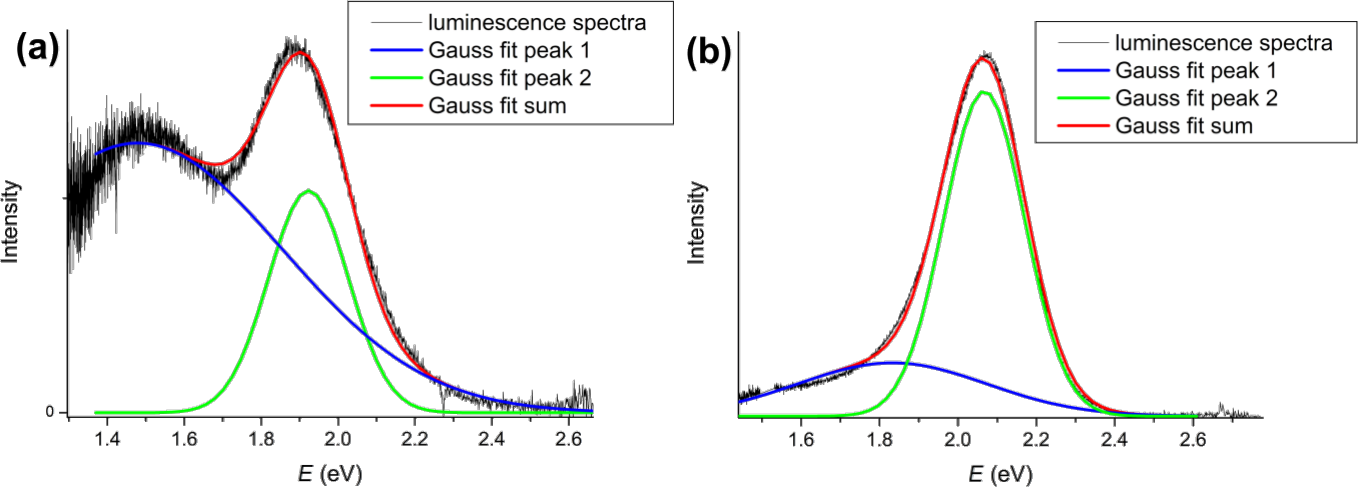
PL spectra of **(a)** non-doped QDs and **(b)** one of the doped QDs deconvoluted into energy coordinates by using two Gaussian functions.

The PL spectra were deconvoluted in energy coordinates by using two Gaussian functions: One is related to the excitonic peak and the other to surface defects (Figure 9a) in case of non-doped sample and to the dopant in case of doped QDs, respectively (Figure 9b).

**Figure 10:**
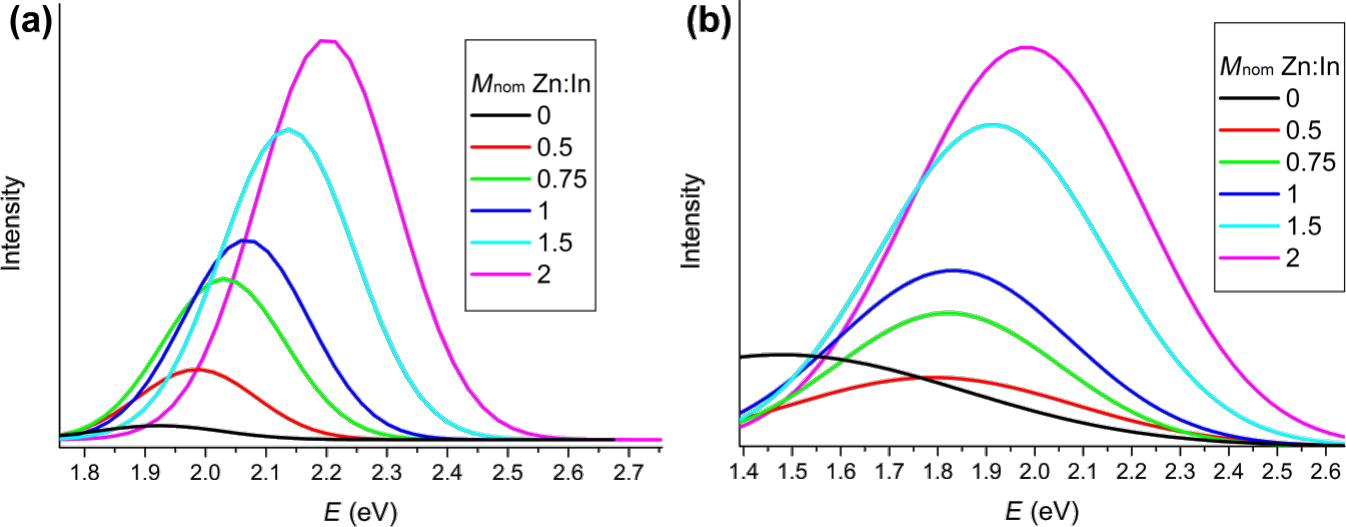
(a) Evolution of the excitonic peak with increasing *M*_nom_; (b) evolution of the defect peak with increasing *M*_nom_.

Figure 10a shows how the excitonic peaks change with increasing *M*_nom_. The peaks shift to larger *E* values (blue shift) and the intensity increases. The same dependence is observed for the Zn peak, too (Figure 10b). Both types of peaks change simultaneously and the distance between their maximum is a constant value (ca. 0.2 eV). In the case of the non-doped sample, the peak related to the surface defects is very broad and is shifted away from the excitonic peak by about 0.4 eV. These facts definitely prove that the tail of luminescence is of a different nature and most probably caused by the doping with Zn atoms.

**Figure 11:**
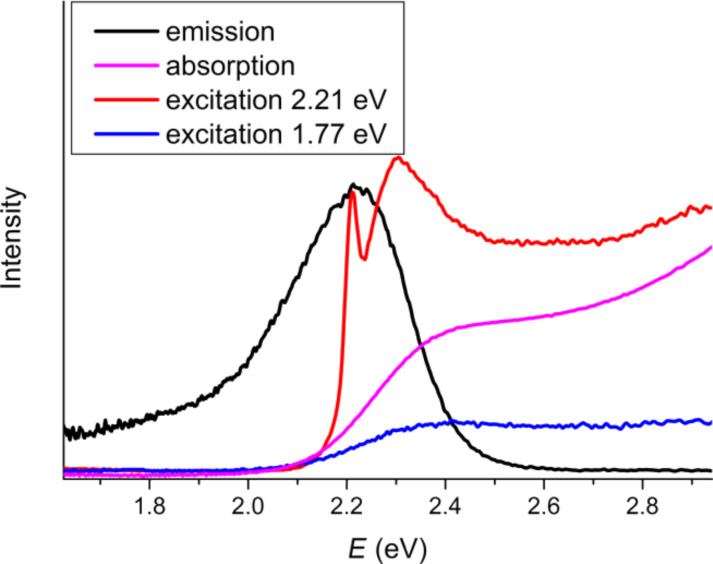
Comparison of typical excitation, PL and UV–vis absorption spectra.

**Figure 12:**
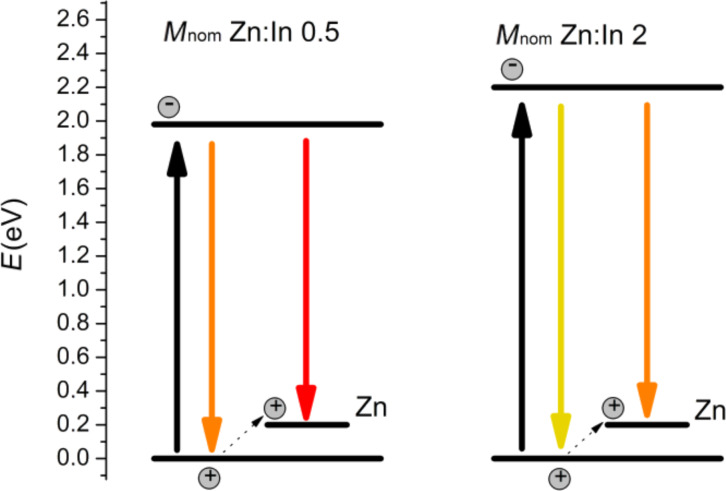
The scheme of PL formation with participation of Zn levels.

The excitation spectra are given in Figure 11. Both types of luminescence are excited together and the maximum of the excitation spectra matches with the maximum of the absorption spectrum. The fact that the Zn luminescence is not excited without excitonic luminescence as well as the fact that the both types of peaks are shifted together and the distance between them is constant could be explained through the doping scheme in Figure 12. This scheme solely depends on the assumption that the Zn levels are pinned in the band gap when the size of the QDs changes. The valence band energy of bulk InP is set to zero. The quantum confinement effect takes place when the size of the particle is less than the exciton Bohr radius [23]. Besides, the strong confinement is fulfilled when the size of the particle is less than the Bohr radius for all types of charge carriers. According to the literature [5] the exciton Bohr diameter for bulk InP is about 21.6 nm, the Bohr diameter of electrons is about 19.6 nm and of holes about 2.0 nm. The mean diameter of the synthesized particles is in the range of 1.5–6.5 nm. Thus, quantum confinement effects for the most particles are extended to the electron levels but not to the hole levels. When the size of particles decreases the band gap increases on the account of moving electron levels. The Zn-based PL originates from an electron in the electron levels and a hole in the Zn level, which skips by excitation from the QDs hole levels. In this case Zn-based PL should be size dependent, differ from the excitonic PL by the same value (the distance between the Zn levels and the hole levels), and should be excited simultaneously with excitonic PL. The fact that the intensity of the defect peak is lower than that of the excitonic peak can be explained with a relatively slow recombination of electrons and holes at the Zn level. The recombination rate is proportional to the overlap of the electron and hole wave functions. For QDs the electron–hole overlap is far greater than in the case that the hole skips on the Zn level. Hence, the recombination rate is higher faster and therefore the luminescence intensity is higher.

**Figure 13:**
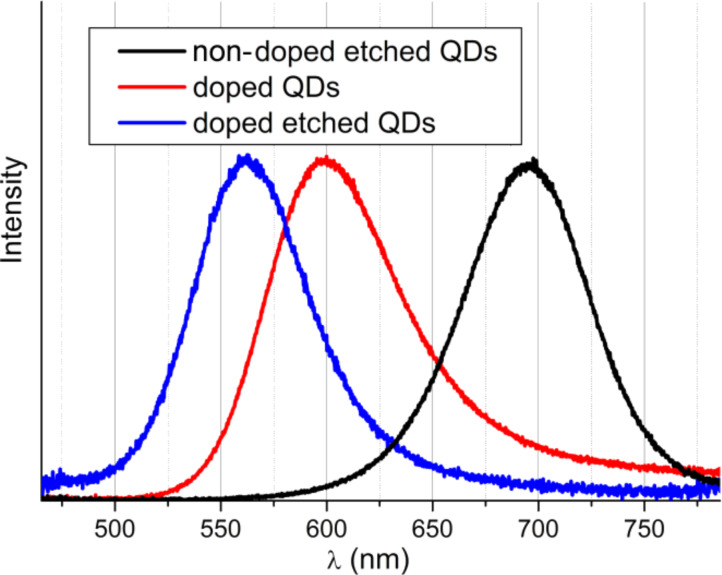
Normalized PL spectra of three samples: non-doped etched with HF, doped with Zn and etched with HF, and doped with Zn non-etched QDs.

The doping process is a process of dopant incorporation inside the structure of the QDs. Thus, it is necessary to prove that the dopant atoms are located inside the InP lattice (and not only at the surface of QDs). The position of Zn could be determined by using photochemical etching with HF. According to the literature [24], during daylight photoetching a significant blue shift of the luminescence maximum is observed, which means that the diameter of the QDs decreases because of a partial dissolution of the QDs. If the Zn atoms are located only on the surface of the QDs, then photoetching should lead to the removal of all Zn atoms and the optical properties should be identical to non-doped photoetched QDs. If the Zn atoms are located inside the core of the QDs, the optical properties should be similar to the optical properties before photoetching. Figure 13 depicts the normalized PL spectra of three samples: non-doped etched, doped etched and non-etched QDs. It can be clearly seen that after photoetching the non-doped sample exhibits a symmetric PL peak shape (without surface defect peak) while the PL peak of the doped sample is asymmetric and possesses a tail in the red region of spectra. At the same time the PL spectrum of the etched doped sample is blue shifted relatively to the non-etched sample, which means that the size of the QDs is decreased and the upper layer is removed, and exhibits the same asymmetric shape as the doped non-etched sample. Thus, we claim that Zn atoms are located inside the core of the QDs. The XRF analysis confirms the presence of a small amount of Zn, *M*_exp_ ≈ 0.06, after etching.

## Conclusion

In summary, we have developed for the first time a phosphine-based synthetic route for Zn-doped InP colloidal QDs over a wide range of Zn doping. We have obtained dependencies of the optical properties on the nominal amount of Zn. We have proposed a growth mechanism of Zn-doped InP quantum dots and have demonstrated that during the synthesis of InP(Zn) QDs two types of processes occur: a covering with a shell of surface Zn-atoms and an incorporation of Zn atoms into the InP lattice. The first process leads to a drastic increase of the PL intensity because of the reduction of phosphorous dangling bonds and is an important preliminary step in the creation of core–shell (with ZnS or ZnS shell) particles. The second process leads to the formation of a Zn level in the InP band gap and to a red shifted tail in the PL spectra. By using photochemical etching with HF, we have confirmed that the Zn dopant atoms are situated inside the InP nanoparticles.

## Experimental

### Synthesis

Colloidal InP QDs with an average diameter of 1.5–6.5 nm were prepared according to a method recently proposed in literature [24] using gaseous phosphine as phosphorous precursor. Indium and zinc precursors were mixed with myristic acid in proportion to indium (3:1 molar ratio) and dissolved in octadecene at 215 °C in neutral Ar atmosphere. *M*_nom_ was varied for each synthesis. The mixture was maintained at this temperature for one hour to remove acetic acid and water. After that, a certain amount of PH_3_ was bubbled through the solution. The growth of QDs continued for 15 min, and then QDs were rapidly cooled and purified. For synthesis we used high-purity argon, PH_3_ (high purity, mixture with argon 1:1), anhydrous indium acetate (In(OAc)_3_, Sigma-Aldrich, 99.9%), zinc acetate dehydrate (Zn(OAc)_2_, Sigma-Aldrich, ≥98%), myristic acid (98%, Fluka). Hexane, acetone, acetonitrile, THF (reagent grade) and octadecene (ODE, 90%) were used as solvents. Rhodamin 6G (laser grade) was used as standard for the determination of the photoluminescence quantum yields (QY).

Right after synthesis hexane was added to the reaction mixture and a white precipitate was removed through centrifugation. According to XRF spectroscopy, this precipitate consists almost of zinc myristate. To purify the synthesized QDs, we carried out the precipitation with a mixture of acetone and acetonitrile. Afterwards, the precipitated QDs were separated by centrifugation and re-dissolved in hexane.

The photochemical etching with HF was performed as described in literature [24] as follows: An aliquot of InP nanocrystals solution dispersed in hexane was mixed with THF and a certain amount of myristic acid was added. The mixture was loaded into a perfluoroethylene vessel and a certain amount of etching mixture (HF in THF 1:10) was added under stirring.

### Characterization

The UV–vis absorption spectra were measured at room temperature with a Varian Cary 50 spectrophotometer in a 1 cm quartz cuvette from 200 to 1100 nm. Photoluminescence (PL) spectra were measured in the same cuvette at room temperature with an Ocean Optics 4000 USB spectrometer calibrated by using a 2600 K W-lamp. Excitation of PL was carried out by using a 405 nm continuous laser LED (40 mW). Powder X-ray diffraction (XRD) patterns were taken on a Rigaku D/MAX 2500 diffractometer using Cu Kα radiation (λ = 1.540598 Å). Transmission electron microscopy (TEM) and electron diffraction (ED) studies were performed using a Tecnai G2 30 UT (LaB_6_) microscope operated at 300 kV with 0.17 nm point resolution and equipped with an EDAX EDX detector. High angle annular dark field (HAADF)-scanning TEM (STEM) studies and EDX mapping were performed using an JEM ARM200F cold FEG double aberration corrected electron microscope operated at 80 kV and equipped with a large solid-angle CENTURIO EDX detector and Quantum EELS spectrometer. XRF spectroscopy was performed with a Bruker M1 Mistral spectrometer, the beam energy was 50 keV. The measurements were performed with Mo filter to diminish the background signal. First, a series of standard samples were prepared in form of ODE solutions containing indium myristate or zinc myristate and the calibration curve was obtained from measurements of standard samples. Both standard samples and QD samples were employed as ODE solutions and sols and placed in polyethylene cuvettes for measurements. The analytical signal was determined as relation of Zn K line integral intensity to In K line integral intensity. Excitation spectra were measured at room temperature with LS-55 Perkin Elmer spectrometer in a 1 cm quartz cuvette in the range of 200–900 nm with 0.5 nm resolution.
